# An exploratory study of public’ awareness about robotics-assisted surgery in Kuwait

**DOI:** 10.1186/s12911-020-01167-1

**Published:** 2020-07-01

**Authors:** Ali Jasem Buabbas, Saad Aldousari, Abrar Abdulmohsen Shehab

**Affiliations:** 1 Department of Community Medicine & Behavioral Sciences, Faculty of Medicine, Kuwait University, P.O. Box 24923, 13110 Safat, Kuwait; 2 Department of Surgery (Urology Division), Faculty of Medicine, Kuwait University, P.O. Box 24923, 13110 Safat, Kuwait; 3 Department of Immunology, Mubarak Alkabeer Hospital, Hawally Health Region, Ministry of Health, Jabriya, Kuwait

**Keywords:** Robotic-assisted surgery, Public, Awareness, Perceptions

## Abstract

**Background:**

The adoption of robotic-assisted surgery (RAS) requires a clear willingness, not only from healthcare organization to operate the robotic system but also from the public that is going to perceive it. This study aims to explore public’s awareness, understanding and their perceptions towards RAS in Kuwait.

**Methods:**

This cross-sectional study used a survey questionnaire that was disseminated on a tablet device to people at their convenience in governmental institutions.

**Results:**

A total of 1087 people agreed to participate in this study. The study results showed that only 36.8% of respondents had heard of RAS and 27.1% knew what RAS is. Moreover, 47.6% of the respondents were uncertain about its safety, while 29.7% thought RAS was safe. The results also showed that 40.9 and 34.4% of respondents thought that RAS is more precise and faster than conventional surgical procedures, respectively, whereas 30.6% feared malfunctioning issues during surgical procedures.

**Conclusion:**

This public survey among a Middle Eastern population reveals lack of awareness and limited understanding of RAS. However, there was a tendency towards believing that RAS may have potential advantages in terms of better outcomes compared to conventional surgical procedures.

## Introduction

Since the approval of the da Vinci® Surgical System (Intuitive Surgical Inc., Sunnyvale, California, USA) by the Food and Drug Administration in the United States of America (USA), the use of robots in surgery has become popular in hospitals worldwide as a method of minimally invasive surgery (MIS). As an evolving approach, robots have been used extensively in Europe and the USA to perform complex surgeries in multiple specialties, including urological, gynecological, cardiothoracic, and colorectal procedures [1, 2]. Previous studies have shown that robotic-assisted surgery (RAS) aids in performing precise surgical procedures with better outcomes and shorter postoperative hospital stays [3–6]. Nevertheless, the widespread acceptance of RAS might be influenced by the public awareness of this technology and the total cost of RAS programs [7]. Most of the previous studies have investigated the attitudes of patients and/or health care staff toward RAS [8–10], while there is little research into public understanding and perceptions [11].

The adoption of RAS requires clear willingness not only from healthcare organization to operate the robotic system but also from the public that is going to perceive it. An international survey study was conducted to explore public perceptions (*n* = 747) of RAS, where 94% of respondents were from the USA [11]. The study showed that most of the respondents (87%) had heard RAS was faster and safer and produced less pain and better outcomes than conventional laparoscopic surgery. However, 55% of the respondents preferred conventional surgical approaches. A previous study found that the most important factors associated with patient decision-making regarding MIS are: Safety, degree of postoperative pain, and recovery time [12]. Furthermore, despite the widespread use of RAS in populations, such as in the USA, public acceptance of this technology still poses a challenge and has been found to be directly related to the public’s educational level and experience with the use of social media [13]. Interestingly, individuals who like to use computer technology are more accepting of the use of advanced healthcare technology, including RAS [14].

Overall, due to the lack of research in the extant literature exploring the awareness and perceptions of the public on RAS, it is important to conduct a research study to fill the knowledge gap in this domain.

In the state of Kuwait, a Middle Eastern country with only two da Vinci® Surgical Systems (Intuitive Surgical Inc.), the first of which was installed in 2013, there is a tendency towards obtaining additional robots into the healthcare system to provide high-quality patient service. However, public awareness and perceptions of RAS in Kuwait are unknown. Therefore, this study aims to explore public awareness, understanding and their perceptions towards RAS, with an emphasis on factors that influence their perceptions.

### Research questions

- ▪ To what extent are people aware of and understand RAS as an option in surgery?
- ▪ What are the public’s perceptions of RAS? If they have experience with RAS, what are their opinions?
- ▪ What are the associations between the social demographics of the participants and other variables in the study, particularly for those with medical backgrounds?

## Materials and methods

### Study design

This cross-sectional study used a survey questionnaire that was electronically developed using Google Docs. This cross-sectional study used a survey questionnaire that was disseminated on tablet devices to people at their convenience in governmental institutions in Kuwait.

### Research instrument: survey questionnaire

The questionnaire’ items were adopted from previous studies [11, 14], with modifications made to suit the research setting and objectives of this study.

Preliminary fieldwork was undertaken to ensure that research instrument is suitable for collecting data and meet the objectives of the study. Thus, content validity of questionnaire’ items was checked by a panel of two surgeons from Kuwait Ministry of Health and two experts in health informatics from Kuwait University. Thereafter, a pilot study was conducted with ten respondents to test the suitability of the items in the questionnaire and amendments were made for items that needed more clarification, such as RAS definitions, and adding one more option to the Likert scale, which was ‘I don’t know’.

The questionnaire comprised 21 items and consisted of four sections (see Additional file 1): (1) demographic data section consists of 5 items; (2) experience with technology (3 items); (3) awareness and understanding (5 items), and perceptions of RAS (5 items); in addition to identify the perceptions of respondents who had experience with RAS (3 items) (if applicable).

Participants of ages less than 21 years were excluded from the study. The questionnaire was available in two languages: Arabic (native language) and English. The Arabic translation was performed by the Translation Office in the Faculty of Medicine at Kuwait University. The study was conducted between December 2018 and June 2019.

### Ethical consideration

Ethical approval was obtained from the Research Committee at the Kuwait Ministry of Health (reference number: 578). An informed consent form was obtained from each participant who agreed to participate in completing the questionnaire.

### Statistical analysis

The data were analyzed using the Statistical Package for Social Sciences (SPSS) Version 25. The data were processed to develop several graphical illustrations for the demographic characteristics of the public, such as frequency tables and charts. The results were calculated for categorical variables and the chi-square test was used to test the associations between these variables. A t-test was used to test for differences in the means of two independent samples. A *p* value ≤0.05 was considered significant. The reliability of the questionnaire items was tested, where Cronbach’s alpha = 0.657.

## Results

### Socio-demographic data

A total of 1087 people agreed to participate in this study; 753 via social media link and 334 out of 350 from governmental institutions using a tablet device, giving a response rate of 95%. A hundred-thirty respondents aged below 21 years old were excluded, leaving 957 respondents: 318 (33.2%) and 639 (66.8%) were males and females, respectively. In total, 899 (93.9%) were natives from Kuwait, and 58 (6.1%) were expatriates living/working in Kuwait. The age ranged from 21 to 70 years old, with a mean of 33.10 ± 9.49 years. The mean age for females was significantly lower than that for males (32.27 ± 8.37 vs. 34.77 ± 11.23, *p* = 0.001). Sixty-three percent of respondents had a bachelor’s degree, and 21.2% had medical or medical-related professions (Table 1).

**Table 1:** Sociodemographic data of the 957 respondents

Education	n	%
High school	37	3.9
Diploma	191	20.0
Bachelor	598	62.5
Postgraduate	127	13.3
Other	4	.4
**Profession**		
Non-medical	627	78.9
Medical-related	104	13.1
Medical	64	8.1
**Hours spent using technology devices per week**		
0–5	220	23
6–11	277	28.9
12–17	179	18.7
≥ 18	281	29.3
**Comfort level in using technology devices**		
Not comfortable	41	4.3
Somewhat comfortable	295	30.8
Comfortable	621	64.9
**Literacy in technology**		
Illiterate	196	20.5
Literate	329	34.4
Competent	432	45.2
**Awareness of RAS**	352	36.8
Internet/social media	**136**	**14.2**
Other	**147**	**78.6**
Not sure	**69**	**7.2**

The results showed there were no significant associations between the understanding of RAS and the age of respondents (*p* = 0.634), hours spent using technology devices (*p* = 0.700), comfort in using technology devices (*p* = 0.148), and/or literacy in technology (*p* = 0.194). However, there was a significant association between the understanding of RAS and the educational level of respondents; the higher their educational level, the more likely the respondent had a good understanding of RAS (*p* = 0.015). Furthermore, there was a significant association between having a medical or medical-related profession and the understanding of RAS (*P* < 0.0005); those who worked in medical or related fields were more likely to understand RAS compared to those who did not (54.0% vs. 26.7%, respectively).

In this study, 40.1% of the respondents with medical or medical-related professions understood RAS, compared to 25.9% of the respondents with nonmedical professions. There was a statistically significant association between having a medical or medical-related profession and the belief that RAS is safe, precise, produces less pain and fewer complications, and understanding of which surgical specialties use it. Also, there was a statistically significant association between having a medical or medical-related profession and fear of intraoperative malfunction, and believing RAS was slower than nonrobotic (laparoscopic and open) surgery (*p* ≤ 0.05). The results revealed no significant association between those respondents and recommending RAS as a surgical option (*p* = 0.076).

### Comfort with technology

Most respondents (77.5%) were experienced with using technology devices such as computers, smartphones, and tablets for 6 to more than 18 h a week, and 65.9% felt comfortable using these technology devices. The majority of the respondents were either literate (34.4%) or competent (45.2%) in using a computer. Computer-literate respondents and those who were comfortable using technology devices believed that RAS surgeons are more skillful than conventional open surgeons, and hospitals that offer RAS are better than those hospitals that do not offer RAS (*p* ≤ 0.05).

### Public awareness, understanding and perceptions

**Fig. 1:**
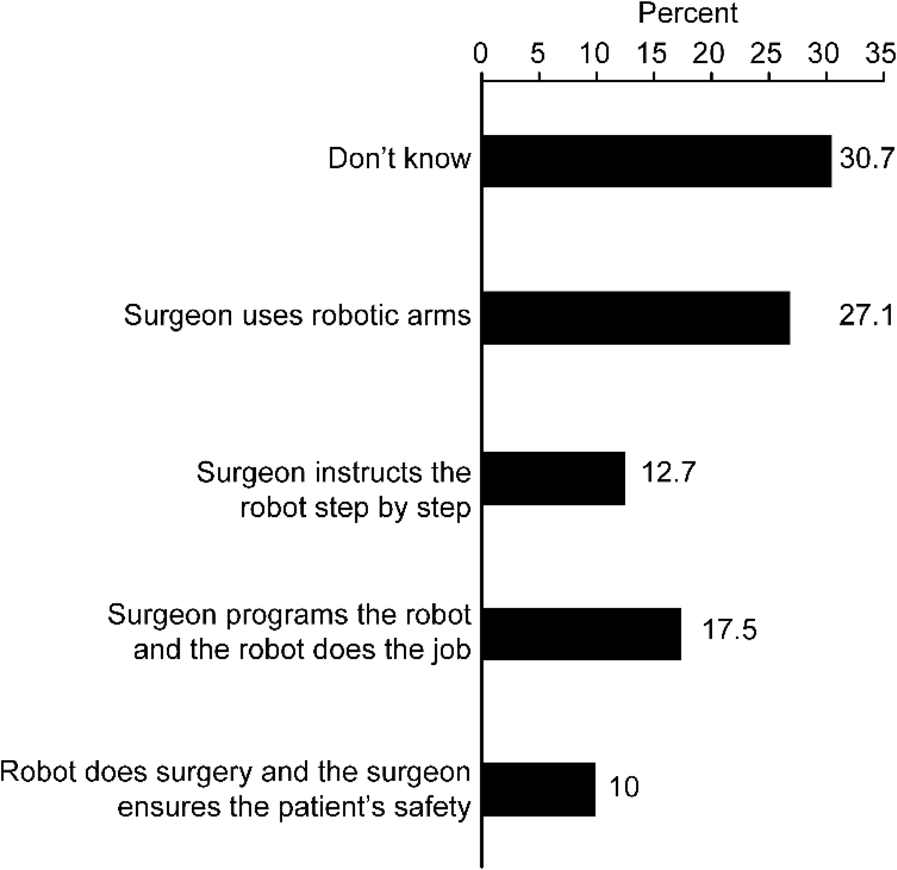
Distribution of respondents’ responses on how RAS performs surgical procedures

Only 36.8% of respondents had heard of RAS, almost half of whom had heard of RAS in social media platforms and/or internet-related sources. Of all respondents, only 27.1% understood that RAS involves a surgeon sitting on a console and control the robot’s movements, 30.7% chose “I do not know”, and the rest of the respondents did not understand RAS and chose wrong definitions for it (40.2%) (Fig. 1). Only 18.8% of the respondents knew that RAS was available in Kuwait. A few of the respondents (6.3%) knew people who had undergone RAS inside or outside of Kuwait. Nearly half of the respondents (47.6%) were uncertain if RAS was safe, while 29.7% thought it was safe and 22.7% thought it was not.

**Fig. 2:**
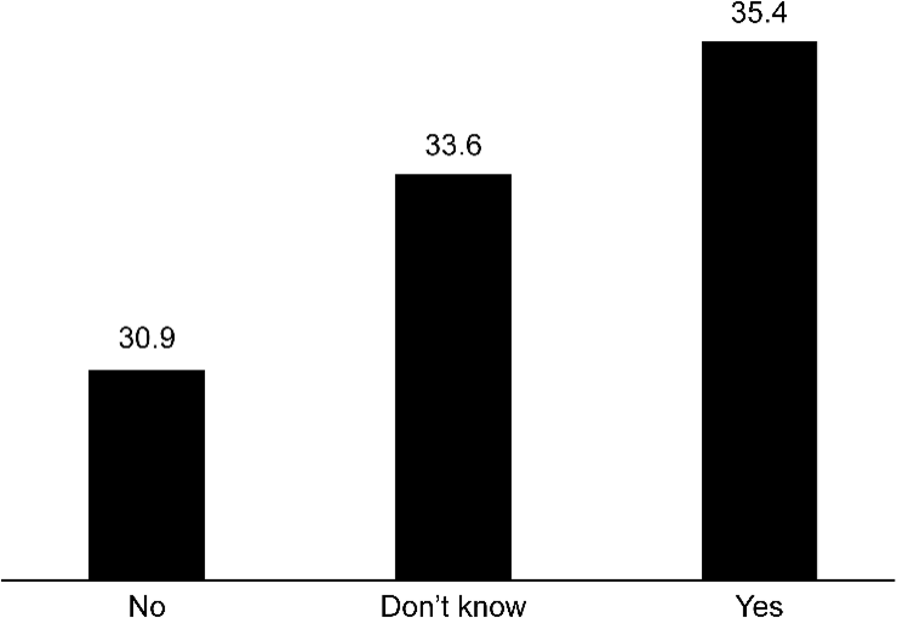
Distribution of respondents’ responses on recommending RAS as a surgical option

When exploring the public’s understanding about which specialties can utilize RAS: 28.4% of the respondents said general surgery, 19.6% cardiac surgery, 15.5% urology, 12.5% neurosurgery, and 7.8% thoracic surgery. While 39.3% of the respondents did not know whether RAS was similar to other forms of surgery, 42.3% thought it is similar to laparoscopy. However, more than one-third of the respondents (35.4%) stated that they would choose RAS if they ever needed surgery in their lifetime, and 30.9% said they would not. (Fig. 2). There was no significant association between choosing RAS as an option for surgery and the respondent’s age, educational level, or comfort level in using technology devices (*p* > 0.05).

**Fig. 3:**
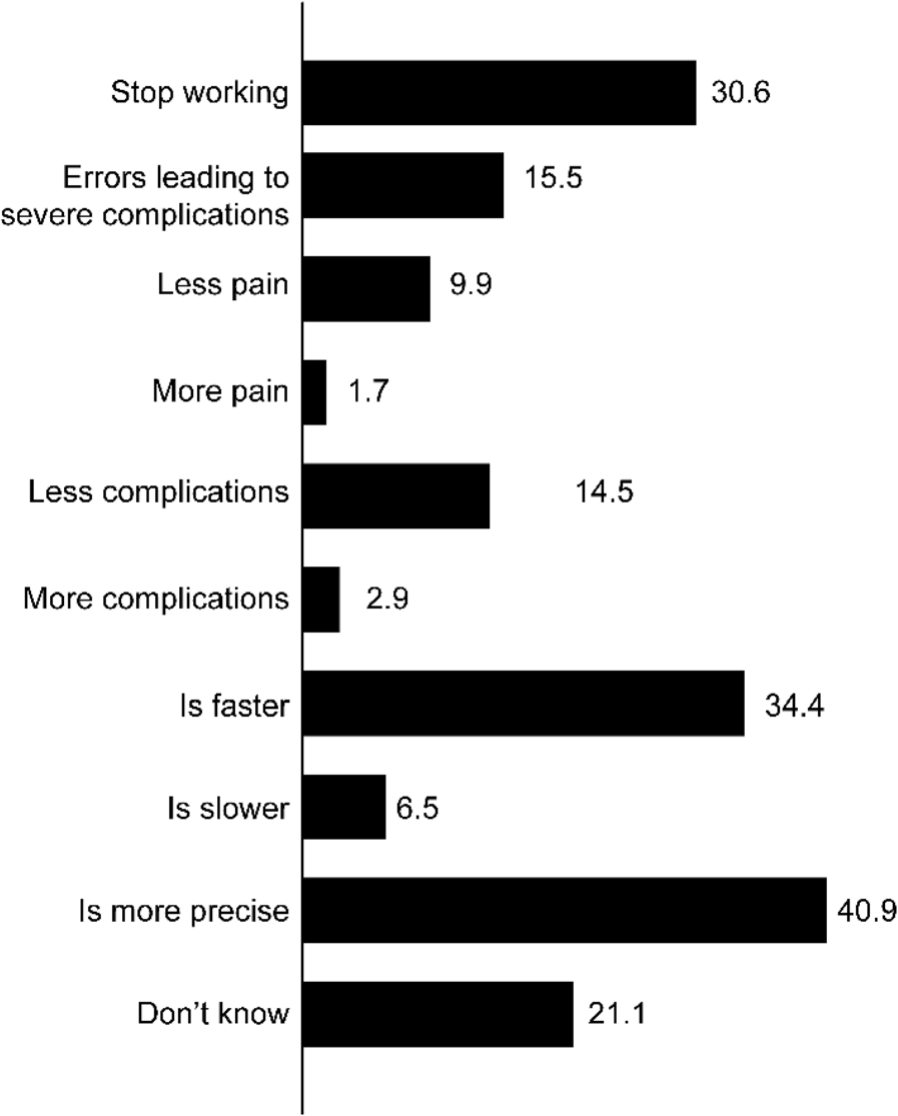
Distribution of respondents’ responses regarding advantages and disadvantages of RAS compared to conventional methods of surgery

The respondents were asked about their perceptions regarding the advantages and disadvantages of RAS compared to conventional surgery (Fig. 3), it was thought among third of the respondents that RAS was more precise and faster compared to conventional surgery. Some respondents feared malfunctioning issues during RAS operations (30.6%) or errors that may lead to severe complications (15.1%). Furthermore, 43.2% of the respondents believed that surgeons who use the robot are more skilled compared to other surgeons, while nearly 52% thought otherwise or were not sure.

More than half of the respondents (51.8%) thought that a hospital that offers RAS is better than other hospitals, only 2.5% thought they were worse, and a minority (21.9%) thought they were similar.

### Perceptions of respondents who had undergone robotic-assisted surgery

Of all the respondents, 22 (2.3%) respondents had undergone RAS themselves: 16 patients (72.7%) had undergone RAS in Kuwait and six patients (27.3%) had undergone RAS elsewhere. Almost all of those patients (21) rated their experience as “good” to “excellent”, with only one patient choosing “I don’t know”. Their choice to undergo RAS as opposed to other forms of surgery was due to their surgeons’ recommendation (40.9%) and believing that they would obtain better surgical results via RAS (31.8%). Of the respondents, 14 of them (63.6%) would recommend RAS to others as a surgical option, including 11 respondents (50%) who had experienced open and laparoscopic surgery in the past.

## Discussion

In general, the more the individual is aware about technology, the more he/she would be willing to accept it. In this study, public awareness towards RAS in Kuwait has been investigated and explored their understanding and perceptions whether/not to accept the advanced technique of surgery using the robot.

### Public awareness, understanding and perceptions

The findings of this study revealed that approximately one-third of Kuwaiti respondents had heard of RAS and < 20% knew it was available in Kuwait. A better understanding of RAS was among 27.1% of respondents and this percentage was significantly associated with respondents who had medical or medical-related professions (21.2%). These findings reflect a significant lack of awareness among the public. When compared to a survey study published previously in which the majority of respondents (94%) were from the USA, 86% had previously heard of RAS, over half (53%) had a background in health care, and 13% were physicians [11]. This could partly explain the wide disparity in public awareness between the former study and the present study, especially since this surgical technology has been popular for many years in the USA healthcare system. Other studies also reported low awareness and understanding of RAS among patients [1, 9, 13].

Although no association was found between comfort in using technology devices and/or literacy in technology and public’ understanding of how RAS procedures are performed, respondents were more likely to think RAS surgeons are more skillful than conventional open surgeons, and hospitals that offer RAS are thought to be better hospitals. This misperception among the public could be related to the false mental association between advanced technology and better outcomes, regardless of their understanding of this technology and how similar it is to other forms of MIS. The findings of this study showed that only 42.3% of respondents thought it was a form of MIS (such as laparoscopy), unlike a previous survey study where public awareness about RAS was higher and the majority (78%) understood that RAS is mostly like laparoscopy [10].

In this study, the findings revealed just over a third of all the respondents would recommend RAS as a surgical option despite believing it to be faster and more precise. However, a third of respondents feared robot malfunction or errors that lead to severe complications, as reported by previous studies [9]. The findings from previous studies showed female gynecological patients did not prefer RAS for their treatment [13, 15]. A survey study was conducted among urologists to determine whether robotic system malfunction occurred in Robotic Assisted Radical Prostatectomy (RARP) and how it was managed [16]. The results revealed that the urologists had faced robotic malfunction, which necessitated rescheduling the case, converting to laparoscopy, or converting to open surgery. Robotic malfunction can happen, and surgeon preparedness should be explained clearly to patients in case such problems occur.

### Perceptions of respondents experienced RAS

Out of a total of 957 respondents, 22 had undergone RAS in the past. Of the respondents that had undergone RAS in the past, almost all of them (21; 95.4%) rated their experience as “good” or “excellent”. Most of them (14; 63.6%) said they would recommend RAS to others, especially 11 of them (50%) who had experience with open, laparoscopic, and robotic surgery in the past. These findings revealed that people who had positive personal experience with RAS, such as less pain and less hospital stay, have influenced their recommendations to others to undergoing RAS when needed.

### Factors influence respondents’ perceptions

In this study, most respondents perceived benefits to RAS but still would not recommend it if surgery is needed in their lifetime. These misconceptions and discrepancies in responses were similarly found in previous studies [11, 13, 16]. This could be explained by the fact that some respondents have limited access to hospitals that provide RAS due to referral-based limitations. In addition, the respondent’s uncertainty regarding RAS safety or the respondents feeling that RAS is unsafe could be another reason.

In this study, there was no significant association between choosing RAS as a surgical option and the respondent’s age, gender, educational level, and comfort level in using technology devices, or having a medical or medical-related profession (*p* > 0.05). In contrast, previous studies found that males were more likely to recommend RAS as a surgical option compared to females [9], and the younger the patient, the more likely he/she will choose RAS [17].

### Research strength and limitations

A strength of this study is the large sample size. However, the use of convenience sampling may not provide a representation of the population of Kuwait. Hence, generalization of this study findings is limited. Another limitation could be that this study was distributed nationally without extending internationally to neighboring countries. Involving neighboring countries could have increased the sample size of the study even more and would have allowed for comparative analysis of a healthcare system with a larger number of RAS programs, such as the kingdom of Saudi Arabia [1]. Furthermore, this study is limited with selection bias; younger people were more likely to participate compared to older people, as demonstrated by the mean age of the study respondents.

## Conclusion

This study concluded that the public has lack of awareness and limited understanding of RAS. However, they think that it could be more precise and offer reduced pain and less complications compared to open surgery or MIS. Fear of robotic system malfunction remained the main factor behind people’s hesitation to undergo RAS. Despite that, the respondents had a positive impression of RAS and would recommend it to others.

### Recommendations

Based on the findings of the study, we set forth recommendations to increase public understanding of RAS.

- ▪ The Ministry of Health in Kuwait should increase the public’s understanding of RAS via campaigns such as using TV screens in hospital waiting areas, distributing brochures, or using social media messages to show the useful aspects of RAS without bias.
- ▪ Surgeons could play a major role in counselling patients about RAS and clarifying how to deal with robotic system malfunction. In addition, surgeons should be encouraged to undertake certified training courses on RAS to ensure patient safety.
- ▪ Conducting research on RAS should be encouraged to investigate the impact of RAS on surgeons, patients, and health care organizations.

## Supplementary information

**Additional file 1.** A survey questionnaire.

## Data Availability

The datasets used and/or analysed during the current study are available from the corresponding author on reasonable request.

## Abbreviations

USA: United States of America
MIS: Minimally Invasive Surgery
RAS: Robot Assisted Surgery
SPSS: Statistical Package for Social Sciences
RARP: Robotic Assisted Radical Prostatectomy
