# Supplementary material for: An exploratory study of public’ awareness about robotics-assisted surgery in Kuwait
Source: BMC Med Inform Decis Mak. 2020 Jul 1;20:140. doi: 10.1186/s12911-020-01167-1 (PMC7329483; doi:10.1186/s12911-020-01167-1)
Supplement: Supplementary file 1 — Additional file 1. A survey questionnaire. [file 12911_2020_1167_MOESM1_ESM.docx]

**Research questionnaire**

**Please tick mark (✓) for the appropriate answer wherever required**

**Demographic data**

1. What is your gender?
2. Male
3. Female
4. What is your age?

…… Yrs

1. What is your nationality?
2. Kuwaiti
3. Non-Kuwaiti
4. What is your educational level?
5. High school
6. Diploma
7. Bachelor
8. Postgraduate
9. Other, please specify……………….
10. If you have a bachelor's degree, can you please select your major from the options below?
11. Medical doctor
12. Medical-related
13. Non-medical

**Experience with technology**

1. On average, how many hours a week do you use computer technology (i.e. computers, I-pods, cell phones)?
2. 0-5 b. 6-11 c. 12-17 d. ≥18
3. How would you categorize your comfort with current technology (i.e. computers, I-pods, cell phones)?

a. Not comfortable b. Somewhat comfortable c. Comfortable

1. How would you rate your computer literacy?

a. Illiterate b. Literate c. Competent

**Awareness and understanding of Robotic-Assisted Surgery (RAS)**

1. Have you ever heard of Robotic-Assisted Surgery?

a. Yes b. No

- If yes, from which resource? -Internet/Social media - Not sure - Other

1. What do you understand from the term Robotic-Assisted Surgery/surgery performed using a robot?
2. The surgeon programs the robot and the robot does the job.
3. The robot does the surgery, while a surgeon stands by to ensure patient’s safety.
4. A surgeon sitting on a console and control the robot’s arms movement.
5. The surgeon instructs the surgical robot step by step.
6. I don’t know.
7. Is Robotic-Assisted Surgery available in Kuwait?

a. Yes b. No c. Uncertain

- If Yes, do you know any patient who had Robotic-Assisted Surgery? a. Yes b. No

1. Do you think Robotic-Assisted Surgery is safe?

a. Yes b. No c. Uncertain

1. In general, which surgical specialties use Robotic-Assisted Surgery? Select all that apply
2. General surgery
3. Orthopaedic surgery
4. Neurosurgery
5. Urology surgery
6. Thoracic surgery
7. Cardiac surgery
8. I don’t know

**Perceptions of Robotic-Assisted Surgery (RAS)**

1. What type of surgery is Robotic-Assisted Surgery most similar to?
2. Traditional open surgery
3. Laparoscopic/minimally invasive surgery
4. Laser surgery
5. Endoscopic surgery
6. I don’t know
7. Would you choose Robotic-Assisted Surgery if it was one of the treatment options for a surgical condition you may have?

a. Yes b. No c. I don’t know

1. What is your perception when you hear the term "Robotic-Assisted Surgery” as a procedure compared to conventional methods of surgery? Select all that apply
2. The procedure is LESS painful than open surgery.
3. The procedure will have LESS complications than open surgery.
4. The procedure is FASTER than open surgery.
5. The procedure is MORE painful than open surgery.
6. The procedure will have MORE complications than open surgery.
7. The procedure is SLOWER than open surgery.
8. Robot malfunction during surgery is a major concern.
9. Robot mistakes causing serious complications is a major concern.
10. Robot can be so accurate it will help the surgeon do a better job.
11. I don’t know
12. Do you think surgeons who use the robot are more or less skilled compared to non-robotic surgeons?
13. More skilled compared to non-robotic surgeons.
14. Less skilled compared to non-robotic surgeons.
15. Similar skills to non-robotic surgeons.
16. Uncertain
17. Do you think hospitals that offer Robotic-Assisted Surgery are better or worse compared to hospitals that do not?
18. Better than hospitals that do not offer
19. Worse than hospitals that do not offer
20. Similar
21. Uncertain

**Robotic-Assisted Surgical patients**

This section is for those who had RAS experience

1. How was your Robotic-Assisted Surgery experience?
2. Excellent
3. Very good
4. Good
5. Bad
6. Very bad
7. Why did you choose this type of surgery? The surgery procedure was ………………..
8. The procedure is safer
9. The procedure is less painful
10. My surgeon recommends this type of surgery
11. The procedure is faster
12. The procedure has better result
13. Would you recommend Robotic-Assisted Surgery to anyone requiring surgery in the future?
14. Yes
15. No
16. Uncertain
